# Magnetically controlled graphene field-effect transistor biosensor for highly sensitive detection of cardiac troponin I

**DOI:** 10.1186/s11671-023-03886-6

**Published:** 2023-08-29

**Authors:** Xiaofeng Zhu, Kangning Cheng, Yue Ding, Huanqing Liu, Shuqi Xie, Yuwei Cao, Weiwei Yue

**Affiliations:** Shandong Province Key Laboratory of Medical Physics and Image Processing Technology, School of Physics and Electronics, Shandong Normal University, Jinan, 250358 People’s Republic of China

**Keywords:** MGFETs, ITO, Aptamer, Sandwich-type complex, CTNI

## Abstract

Herein, we have constructed a magnetic graphene field-effect transistor biosensor (MGFETs) for highly sensitive detection of cardiac troponin I (CTNI). Graphene films transferred to ITO conductive glass as conductive channels. CTNI aptamer was immobilized onto the graphene film via 1-pyrene-butanoic acid succinimidyl ester (PBASE) to capture CTNI. Magnetic nanobeads (MBs) modified with CTNI antibody were added to the reaction chamber to form an aptamer/CTNI/antibody/magnetic nanobeads sandwich-type complex. We found that the magnetic force exerted on the complex leads to an impedance change of the graphene film. The reason for this result is that the magnetic field exerts an influence on the MBs, causing CTNI aptamer strand to bend, resulting in a change in the distance between the double conductive layers of the graphene film surface and the test solution. With periodic sampling integration, different concentrations of CTNI can be detected with high sensitivity. Due to the stringent recognition capability and high affinity between the CTNI aptamer and CTNI, MGFETs have the potential to detect various types of proteins. Furthermore, MGFETs also have the potential to be utilized for the detection of DNA or specific cells in the future.

## Introduction

Based on statistical data from the World Health Organization (WHO), cardiovascular diseases have emerged as the primary cause of global mortality, with acute myocardial infarction (AMI) being the deadliest among various cardiovascular conditions [1, 2]. The prompt detection of myocardial infarction can greatly improve the efficiency of clinical treatment. Presently, the clinical diagnosis of acute myocardial infarction (AMI) predominantly depends on the observation of alterations in electrocardiogram (ECG) waveforms [3–5]. Nevertheless, certain patients experiencing AMI may not display abnormalities in their ECG, and the sensitivity of ECG testing is limited.

To address these limitations, various cardiac biomarkers have been developed for the diagnosis of acute myocardial infarction (AMI). These include cardiac troponin I (CTNI), CK-MB, cardiac troponin T (CTNT), and myoglobin [6, 7]. CTNI is widely acknowledged as the “gold standard” for diagnosing AMI among these cardiac biomarkers [8, 9]. This is due to the fact that CTNI levels specifically reflect heart muscle cell damage, distinguishing it from patients with skeletal muscle diseases. In individuals without cardiac injury, the typical level of CTNI is usually below 0.2 ng/mL. However, when there is damage to cardiomyocytes, CTNI levels tend to range from 0.2 to 2 ng/mL. CTNI levels exceeding 2 ng/mL are associated with an elevated risk of sudden cardiac death [10]. Therefore, the importance of a sensitive and rapid CTNI test cannot be emphasized enough in effectively preventing sudden death in cases of AMI.

Mainly, CTNI detection methods include enzyme-linked immunosorbent assay [11], radioimmunoassay [12], electrochemical analysis [13], colorimetric assay [14], fluorescence immunoassay [15], surface plasmon resonance [16], etc. Nonetheless, these techniques come with high costs, lengthy testing times, and a requirement for higher operator expertise. As a result, biosensors have garnered increased interest for CTNI detection, with options such as electrochemical biosensors, surface plasmon resonance immunosensors, and field-effect transistor biosensors (FETs). Among these alternatives, FETs have gained considerable attention due to their remarkable advantages, including real-time detection capability and high sensitivity [17].

Graphene is a single atomic layer of two-dimensional crystals formed by carbon atoms linked by SP2 hybridization. It has good electron mobility and many oxygen-containing groups that can be used to anchor DNA, proteins, and other biomolecules [18]. Many researchers have investigated the interaction between DNA, proteins, and small molecules utilizing graphene field-effect transistors (FETs), which are created by combining graphene with the principles of field-effect transistors [19, 20].

In conventional graphene field-effect transistor biosensors (GFETs), applying a gate electric field results in the formation of double conductive layers between the electrolyte solution and the graphene film. Researchers have constructed a capacitive model of a GFETs [21]. The gate electrode applies voltage to the Ag/AgCl electrode, controlling the directional movement of charge carriers between the conductive solution and the graphene film, thereby forming double conductive layers. The magnitude of the current between the graphene field-effect transistors is dependent on the carrier concentration and the magnitude of the applied gate voltage [22–24]. The detection concentration GFETs has now reached the nM level.

However, although these techniques have reached extremely high sensitivity through semiconductor analyzers, they are still costly and complicated to use. Additionally, the application of gate voltage through the insertion of Ag/AgCl electrodes into the solution may affect the concentration of the solution, and the gate voltage may cause structural damage to the biomolecules to be measured [25–27].

Therefore, researchers have been exploring alternative methods to improve the performance of GFET biosensors, such as using non-covalent functionalization with biomolecules, which can improve the sensitivity and prevent damage to the biomolecules [28–30]. Other techniques, such as using nanomaterials to modify the graphene film, have also shown promise in enhancing the performance of GFET biosensors [31–33]. These techniques have the potential to evolve into biosensor platforms that can facilitate rapid clinical diagnosis of acute myocardial infarction and enable effective treatment initiation in the future.

Herein, we propose a novel approach for CTNI detection using magnetic graphene field-effect transistor biosensors, which eliminates the need for an Ag/AgCl electrode to provide the gate voltage. Firstly, we transfer graphene films onto glass substrates with two indium tin oxide (ITO) electrodes to establish conductive channels [28]. The graphene films are subsequently functionalized with CTNI aptamers through 1-pyrenyl butyric acid succinimidyl ester (PBASE). To modify the CTNI antibodies, we employ magnetic nanobeads (MBs), which form a sandwich-type immune complex of aptamer/CTNI/antibody with MBs. The reason for the sandwich-like structure is to introduce magnetic beads as a medium for MGFETs, enabling the external magnetic field to control the beads and thereby regulate the resistance changes of the graphene sensor. Antigens and antibodies can bind together through a process called agglutination. Periodic impedance variations of GFETs are achieved by applying magnetic field to MGFETs. The conductivity of GFETs is dependent on the concentration of CTNI. To detect the impedance change of CTNI, we have designed and fabricated the corresponding detection devices in our laboratory.

## Methods

### Materials and instrument required

The conductive glass with ITO electrodes was sourced from Hua Nan Xiang Cheng Ltd. Troponin I and mouse anti-troponin I monoclonal antibodies were obtained from Shanghai Linc-Bio Science CO., LTD. 1-Pyrenebutyric acid N-hydroxysuccinimide ester (PBASE) was acquired from Shanghai Aladdin Biochemical Technology Co., Ltd. 1-Ethyl-3-(3-dimethylaminopropyl)-carbodiimide-hydrochloride (EDC) and N-hydroxy succinimide (NHS) were purchased from Sigma-Aldrich. The troponin I aptamer sequence used, (5-NH2-CGT GCA GTA CGC CAA CCT TTC TCA TGC GCT GCC CCT CTT A-3′-6-FAM), was purchased from Sangon Biotech (Shanghai) Co., Ltd. Carboxylated ferric oxide nanoparticles were obtained from Nanjing XFNANO Materials Tech Co., Ltd. Dimethyl sulfoxide (DMSO) was purchased from Sangon Biotech (Shanghai) Co., Ltd. Sodium dodecylbenzene sulfonate (SDS) and sodium dodecyl sulfate phosphate-buffered saline (PBS, P5368-10PAK; pH 7.4) were obtained from Sigma-Aldrich (Shang-Hai, China). Bovine serum albumin (BSA) was purchased from Shanghai Linc-Bio Science CO., LTD. To evaluate the quality of graphene, a Raman microscopic system (SPEX-1403, SPEX) was utilized. High-magnification transmission electron micrographs (TEM) were captured using a JEOL JEM 2100F instrument.

### Preparation of antibody-functionalized magnetic nanobeads

To obtain a uniformly dispersed magnetic bead solution, the purchased carboxyl-modified magnetic nanobeads were sonicated for 15 min at room temperature. The carboxyl groups on the magnetic nanobeads were activated by adding 5 µL of 0.2 µM NHS and 5 µL of 0.2 µM EDC to 500 µL of 1 mg/mL magnetic nanobeads. Tchenka et al. [29], followed by the addition of PBS to make the total volume 1 mL. The reaction was allowed to proceed for 15 min.

In order to deposit the magnetic nanoparticles at the bottom, a permanent magnet was positioned beneath the magnetic nanoparticle test transistor, and the supernatant was removed using a pipette. The resulting pellet was then resuspended in 1 mL of PBS buffer at pH 7.4.

To functionalize the magnetic nanobeads, 10 µL of mouse anti-troponin I monoclonal antibodies with a concentration of 1 mg/mL were added to the activated magnetic nanobead solution prepared with NHS and EDC. The mixture was incubated at room temperature for 2 h. Following this, 10 µL of 10% bovine serum albumin (BSA) was introduced into the suspension and incubated for 1 h to block any potential residual sites on the nanobeads.

The fixed magnetic nanobeads were obtained by using a permanent magnet underneath the transistor to hold them in place, followed by aspirating the upper layer of unreacted chemicals using a micropipette. The resulting pellet was resuspended in 1 mL of PBS (pH 7.4) supplemented with 1.0% BSA and stored at 4 °C. [29].

### Preparation of magnetically controlled graphene field-effect transistor biosensors

**Fig. 1:**
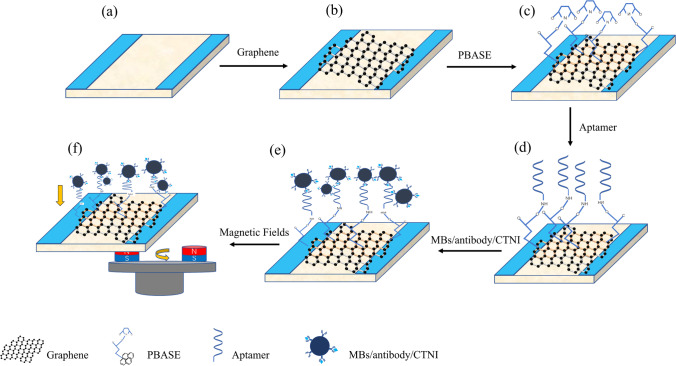
Construction of magnetic graphene field effect transistor. **a** ITO conductive glass. **b** Graphene film grown by chemical vapor deposition. **c** Functionalization of graphene by PBASE. **d** Immobilization of probe aptamer via PBASE. **e** Antibody functionalized magnetic nanobeads and CTNI are added to form sandwich type immune complexes. **f** Applying a periodic magnetic field to a GFETs

The cleaned graphene film was transferred onto a glass substrate with two ITO electrodes, serving as a conductive bridge between the two ITO electrodes (Fig. 1a). The MGFETs utilized the two ITO electrodes as the source and drain. The glass reaction chamber was glued to the surface of the graphene film with violet gel to contain the solution to be measured (Fig. 1b). 10 mM solution of PBASE dissolved in DMSO was injected into the pre-prepared MGFET biosensor reaction chamber and incubated for 2 h to allow PBASE to immobilize on the graphene surface (Fig. 1c). Then, the surface was washed with deionized water and DMSO to remove any unbound PBASE from the graphene film. Next, the –NH2 modified CTNI aptamer was added to the reaction chamber to allow for full binding of the CTNI aptamer to PBASE (Fig. 1d). After the reaction, the surface was washed with PBS solution containing 0.2% SDS, followed by rinsing with PBS solution at pH 7.4 to remove any unbound CTNI aptamer. Subsequently, different concentrations of CTNI were added to the reaction chamber of the GFETs using a standard liquid dispenser. MBs, modified with CTNI antibodies, were then added to form sandwich-type immune complexes (Fig. 1e). Following a 30-min reaction, the magnetic nanobeads were washed 3 times with PBS to remove any excess magnetic nanobeads. Finally, permanent magnets were fixed to a rotating motor to apply a periodic magnetic field to the back of the GFETs (Fig. 1f).

**Fig. 2:**
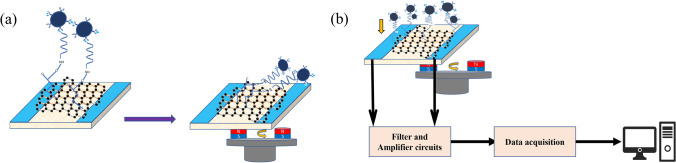
**a** Magnetic beads are moved down by the magnetic field. **b** Flowchart of detection system magnetic beads are moved down by the magnetic field

Through the magnetic force between magnetic beads (MBs) and an externally applied magnetic field, the distance between MBs/aptamer conjugates and the graphene film can be dynamically adjusted. This modulation directly influences the conductivity properties of the graphene film, thereby affecting the impedance of the magnetic field effect transistors (MGFETs) with a dual conductive layer (Fig. 2a). In our laboratory, we constructed a testing system including a filter circuit, an amplifier circuit, a data acquisition circuit, and a LabVIEW upper computer display (Fig. 2b).

## Results and discussion

### Characterization of MGFETs

**Fig. 3:**
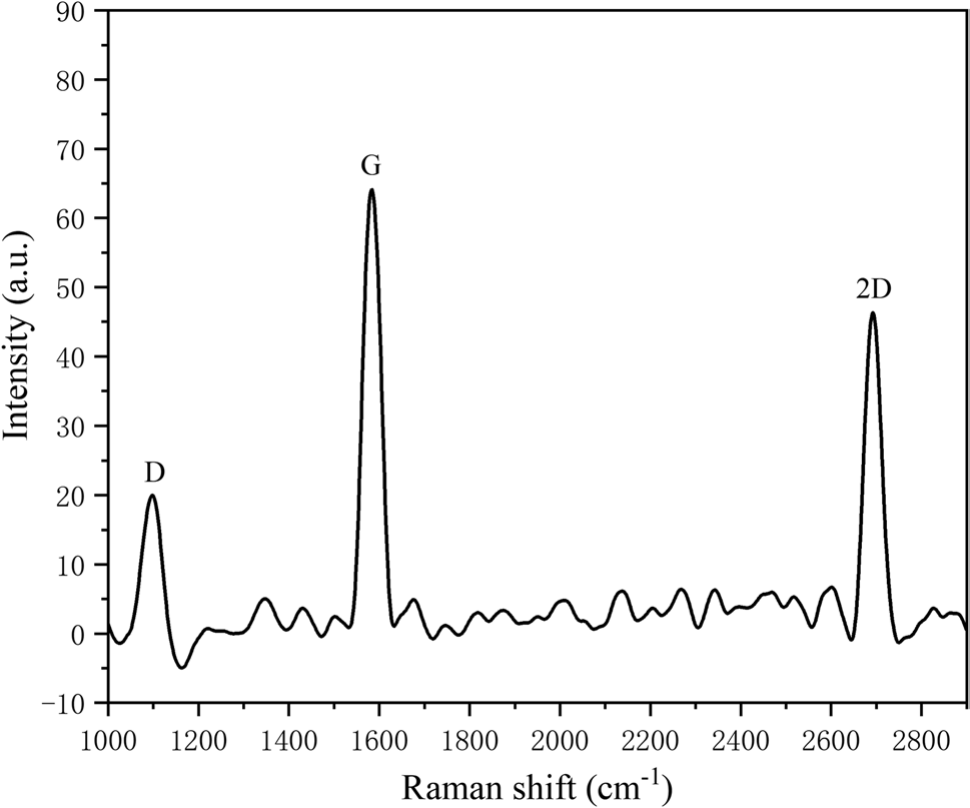
Raman broad spectrum of graphene layers

The transferred graphene was characterized using Raman spectroscopy (Fig. 3). The Raman spectrum indicated the presence of the three characteristic peaks of graphene, signifying the successful transfer of graphene onto the ITO conductive glass [20]. Importantly, the low defect density of the transferred graphene is indicated by the ratio of the D-peak to G-peak intensity (*I*_D_/*I*_G_) and the presence of a small D-peak. Furthermore, the intensity of the 2D-peak of single-layer graphene was more significant than that of the G-peak, while the half-peak width of the 2D-peak gradually increased and shifted to a higher wave number as the number of layers increased. These observations led us to conclude that the transferred graphene films were multilayer films [30–32].

**Fig. 4:**
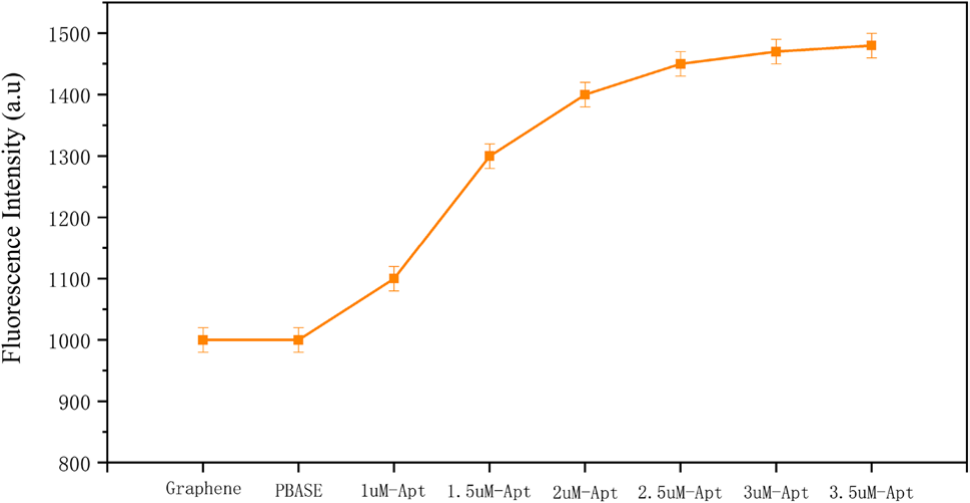
Probe aptamer fluorescence intensity detection on MGFETs. Error bar represents the standard deviation of 5 independent analysis

Notably, graphene cannot bind to cardiac troponin I (CTNI). Alternatively, one end of PBASE can be linked to graphene through *π*–*π* stacking, while the other end can bind to the 5′-NH2 labeled aptamer. We labeled the 3′ end of the CTNI aptamer with a fluorescent group to verify the firm attachment of the aptamer to the graphene film. The (Fig. 4) showed that the aptamer was successfully linked with the graphene film. Increasing the aptamer concentration influenced the fluorescence intensity. A plateau was reached when the aptamer concentration was about 3 µM, suggesting saturation. As such, subsequent experiments used an aptamer concentration of 3 µM.

### Characterization of antibody-functionalized magnetic nanobeads

**Fig. 5:**
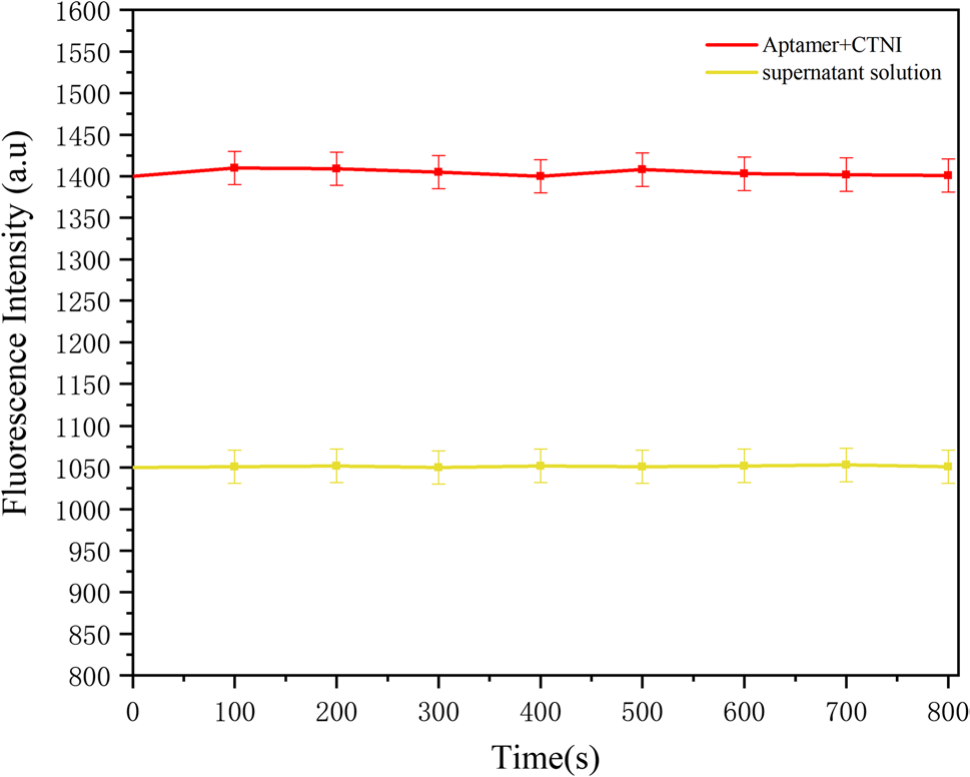
Fluorescence intensity of immune complexes and supernatant solution fluorescence intensity detection

**Fig. 6:**
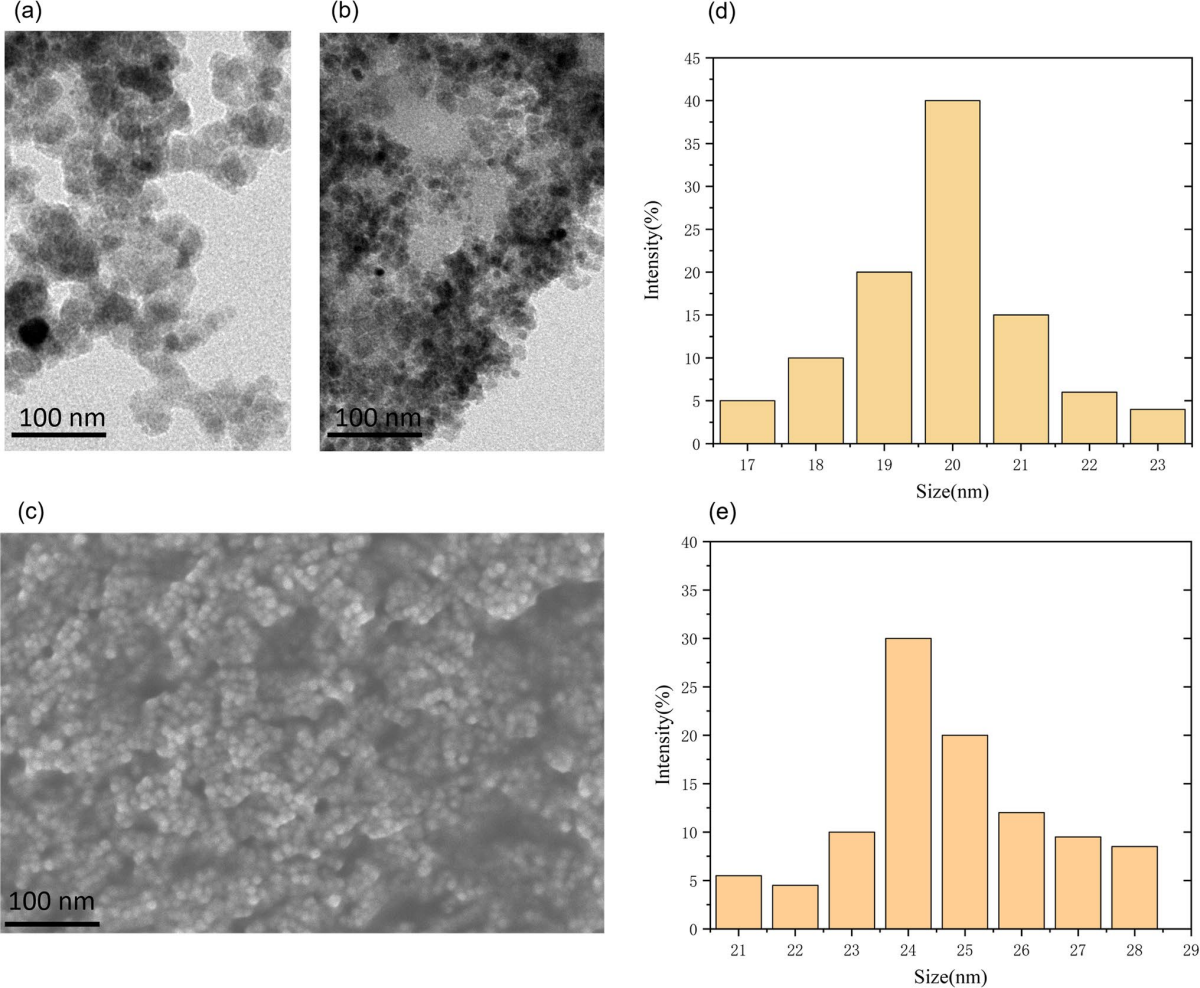
**a** TEM of MBs. **b** TEM of MBs/CTNI antibody conjugates. **c** TEM of CTNI antibody modified magnetic beads on MGFET. **d** Size distribution of magnetic beads. **e** Size distribution of CTNI antibody modified magnetic beads

Carboxyl-modified magnetic nanobeads were activated by NHS and EDC and utilized as a capture probe for binding to CTNI antibodies. The nanobeads were used to form aptamer/CTNI/antibody/MBs sandwich-type immune complexes. Tubes containing 10 µL of CTNI aptamer at a concentration of 2 µM and 100 µL of 10 µM CTNI were incubated for 2 h at room temperature. A fluorescence detection system detected a fluorescence intensity of about 1400 A.U. The mixture of magnetic nanobeads and CTNI antibody binding, which was completed by the aforementioned experiment, was then taken out (100 µL) and added to the test tube. The solution was incubated for 1 h and then separated using a magnetic precipitation method. A part of the supernatant solution was tested using the fluorescence detection system for detection. The results shown in (Fig. 5) indicated a fluorescence intensity of approximately 1050 A.U. Only the fluorescent substance was added to the aptamer to eliminate any interference and enable the formation of sandwich-type immune complexes such as MBs/antibody/CTNI/Aptamer. Due to the specific binding of antibody and antigen, as well as the specific binding of the aptamer to CTNI, a sandwich-type immune complex like MBs/antibody/CTNI/Aptamer was formed in the solution. The complex was deposited at the bottom of the test tube by the magnetic force. The fluorescence intensity of the supernatant was observed to decrease by the fluorescence detection system (Fig. 5), which indicated the successful binding of magnetic nanobeads and CTNI antibodies. The morphology of the MBs and MB/CTNI antibody conjugates was characterized using TEM (Fig. 6a and b). TEM observation of the changes in antibody-modified magnetic beads on MGFETs is shown in Fig. 6c. The size distribution of MB and antibody-modified MB are shown in Fig. 6d and e.

### Analysis of magnetic field intensity

**Fig. 7:**
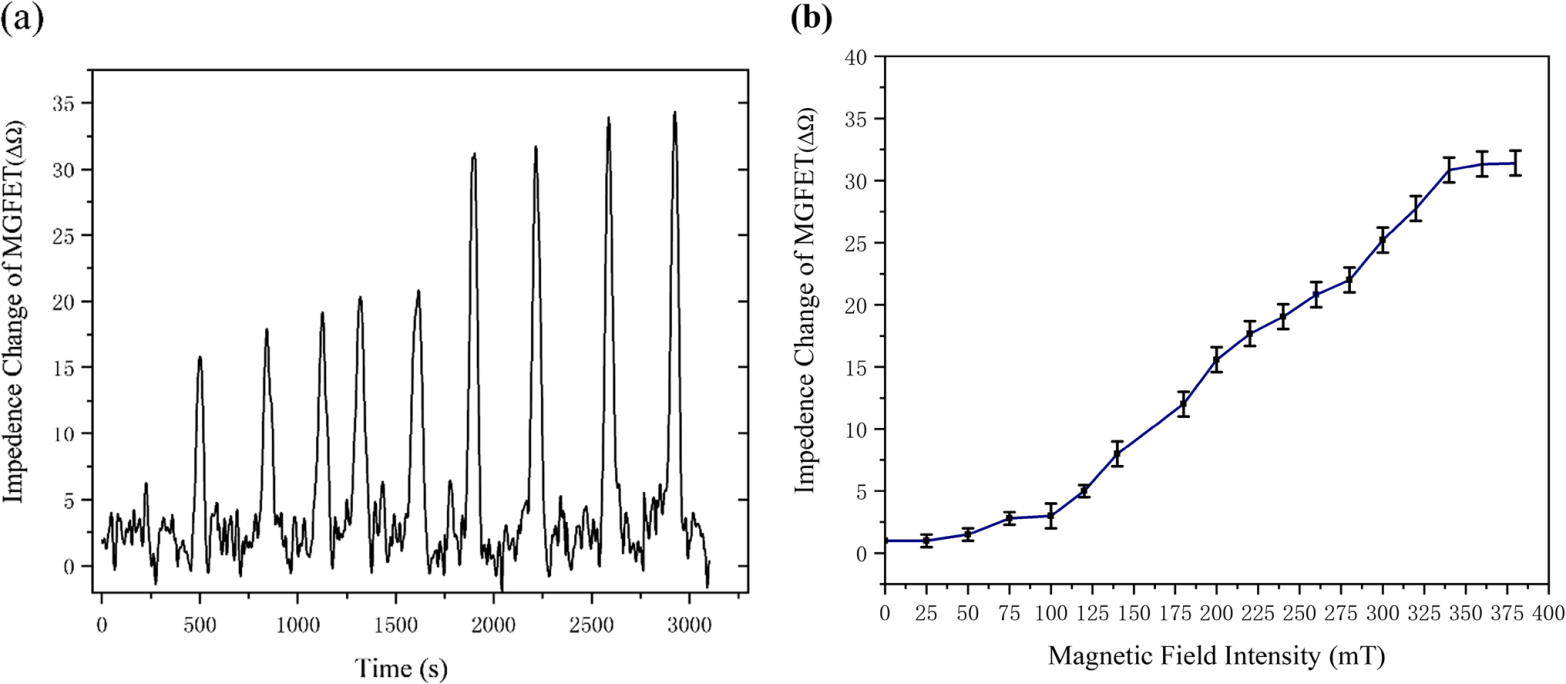
**a** Impedance of MGFETs under a varying magnetic field intensity in the time domain. **b** Relationship between impedance of MGFETs and intensity of the magnetic field. Error bar represents the standard deviation of 5 independent analysis

After the linkage of the CTNI aptamer to the graphene, different concentrations of CTNI solution were added to the reaction chamber of the magnetically controlled MGFET, followed by the addition of an antibody-modified magnetic bead solution for 30 min. The reaction chamber of the MGFET was then washed with PBS to remove any unbound magnetic nanobead and antibody complexes. A permanent magnet was placed at the back of the MGFET to apply a periodic magnetic field, and the impedance change of the MGFET was detected using a laboratory-made device (Fig. 7a).

In conventional GFETs, the gate electrode applies voltage to the Ag/AgCl electrode, controlling the directional movement of charge carriers between the conductive solution and the graphene film. In MGFETs, the CTNI aptamer can be considered as an elastic model. By applying a periodic magnetic field, the bending degree of the CTNI aptamer chain can be altered, leading to a change in the distance of the double conductive layer between the graphene film and the electrolyte solution. This results in carrier movement and subsequently alter the conductivity between the drain and the source of the MGFET. The variation in the impedance of the MGFET with magnetic field strength was divided into three components (Fig. 7b). When the magnetic field strength is less than 100 mT, the support force of the aptamer chain exceeds the magnetic field force, which does not alter the distance between the double conductive layer and hence does not impact the impedance of the MGFET. When the magnetic field strength is between 100 and 330 mT, the magnetic field force is sufficient to bend the aptamer chains, leading to a change in the surface of the graphene and electrolyte solution, which results in a modification of the impedance of the MGFET. When the magnetic field strength is greater than 330 mT, the bending of the aptamer chain reaches its maximum and therefore, the MGFET no longer responds to magnetic field changes, and the impedance of the MGFET remains stable.

### Detection of CTNI

**Fig. 8:**
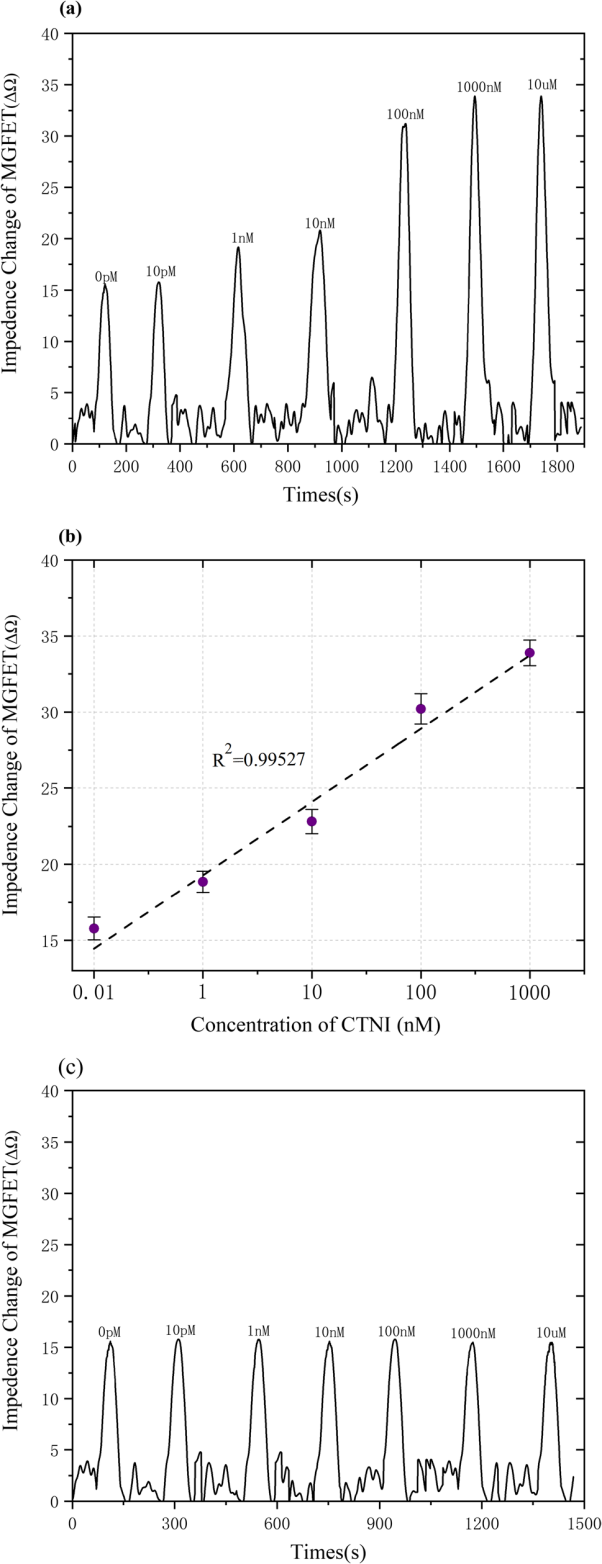
**a** Time domain of impedance fluctuations with different CTNI concentrations. **b** Impedance responses of MGFETs were observed by adding different concentrations of CTNI. Five independent experiments were conducted for each concentration, and the standard deviation was represented by error bars. **c** Impedance changes of MGFETs without the addition of CTNI antibody

The impedance variation of MGFETs with respect to CTNI concentration at a fixed magnetic field strength was measured to determine the sensitivity and detection limit of the MGFETs (Fig. 8a). The MGFETs' impedance change signals were accumulated through the use of a magnetic field and the sampling integration method to reduce noise, and the impedance of MGFETs correlated positively with the concentration of CTNI. (Fig. 8b) The high sensitivity of MGFETs in this study can be attributed to two main factors: firstly, the interaction between the magnetic beads and the applied magnetic field caused bending of the CTNI aptamer chain, leading to a change in the distance between the double conductive layers and a significant impedance variation at the ITO electrode ends. Secondly, we employed periodic magnetic fields to induce periodic impedance changes in MGFETs. By using periodic sampling integration, we effectively reduced the impact of noise and improved the signal-to-noise ratio. In the absence of CTNI antibodies, the magnetic beads do not form sandwich-type immune complexes as they are not modified by CTNI antibodies. Therefore, changing the CTNI concentration does not result in significant changes in the impedance response of the sensor, as shown in the (Fig. 8c).

### Specificity of MGFETs

**Fig. 9:**
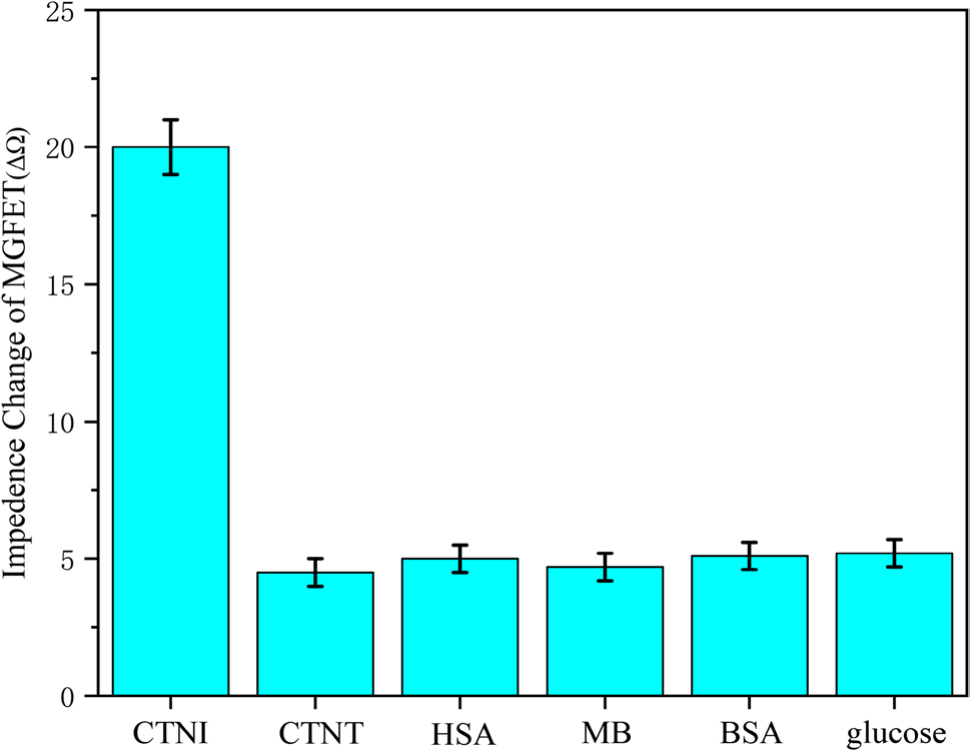
Results based on the detection of CTNI and various non-specific proteins by MGFETs. Five independent experiments were conducted for each biomarker, and the standard deviation was represented by error bars

We evaluated the specificity and selectivity of MGFETs by detecting various types of proteins. Cardiac troponin T (CTNT), human serum albumin (HSA), CK-MB, bovine serum albumin (BSA), and glucose were added to test the specificity of MGFETs, and the different types of proteins were tested alongside CTNI under the same experimental conditions [34, 35]. The experimental results are presented in Fig. 9. The CTNI aptamer exhibits high specificity for binding to CTNI. In contrast to other cardiac biomarkers, which show low binding affinity to the CTNI aptamer, the MGFETs demonstrate excellent specificity for the detection of CTNI.

## Conclusions

To summarize, a graphene-based MGFETs design has been successfully developed as a biosensor for the detection of CTNI. The structure involves laying ITO films on both ends of a glass sheet to serve as the drain-source of the FETs. Then, by transferring a graphene film in the middle, the graphene film was functionalized with an immobilized CTNI aptamer to capture CTNI in solution. A sandwich-type immune complex was formed with CTNI antibody-modified magnetic nanobeads. Finally, a magnetic field was applied to the GFETs by a permanent magnet immobilized under the ITO conductive glass. To reduce noise interference, we utilized the method of periodic sampling and integration to improve the signal-to-noise ratio, achieving high sensitivity detection of CTNI with a detection limit that can reach 10 pM. Our biosensor provides a promising alternative to conventional Ag/AgCl-electrode-based biosensors. Its use of a magnetic field instead of an electric field to control the GFETs not only enables damage-free detection of the biomolecules but also reduces the cost and complexity of the overall detection system.

## Data Availability

All data generated or analyzed during this study are included within the article.
